# Molecular Characterization of *Staphylococcus aureus* Isolated from Bovine Mastitis and Close Human Contacts in South African Dairy Herds: Genetic Diversity and Inter-Species Host Transmission

**DOI:** 10.3389/fmicb.2017.00511

**Published:** 2017-04-06

**Authors:** Tracy Schmidt, Marleen M. Kock, Marthie M. Ehlers

**Affiliations:** ^1^Allerton Provincial Veterinary Laboratory, KwaZulu-Natal Department of Agriculture and Rural DevelopmentPietermaritzburg, South Africa; ^2^Department of Medical Microbiology, University of PretoriaPretoria, South Africa; ^3^Tshwane Academic Division, National Health Laboratory ServicePretoria, South Africa

**Keywords:** *Staphylococcus aureus*, bovine mastitis, PFGE, MLST, inter-species transmission

## Abstract

*Staphylococcus aureus* is one of the most common etiological agents of contagious bovine mastitis worldwide. The purpose of this study was to genetically characterize a collection of *S. aureus* isolates (bovine = 146, human = 12) recovered from cases of bovine mastitis and nasal swabs of close human contacts in the dairy environment. Isolates were screened for a combination of clinically significant antimicrobial and virulence gene markers whilst the molecular epidemiology of these isolates and possible inter-species host transmission was investigated using a combination of genotyping techniques. None of the isolates under evaluation tested positive for methicillin or vancomycin resistance encoding genes. Twenty seven percent of the bovine *S. aureus* isolates tested positive for one or more of the pyrogenic toxin superantigen (PTSAg) genes with the *sec* and *sell* genes predominating. Comparatively, 83% of the human *S. aureus* isolates tested positive for one or more PTSAg genes with a greater variety of genes being detected. Genomic DNA macrorestriction followed by pulsed-field gel electrophoresis (PFGE) of the bovine isolates generated 58 electrophoretic patterns which grouped into 10 pulsotypes at an 80% similarity level. The majority of the bovine isolates, 93.2% (136/146), clustered into four major pulsotypes. Seven sequence types (ST) were identified among the representative bovine *S. aureus* isolates genotyped, including: ST8 (CC8), ST97 (CC97), ST351 (CC705), ST352 (CC97), ST508 (CC45), ST2992 (CC97) and a novel sequence type, ST3538 (CC97). Based on PFGE analysis, greater genetic diversity was observed among the human *S. aureus* isolates. Bovine and human isolates from three sampling sites clustered together and were genotypically indistinguishable. Two of the isolates, ST97 and ST352 belong to the common bovine lineage CC97, and their isolation from close human contacts suggests zoonotic transfer. In the context of this study, the third isolate, ST8 (CC8), is believed to be a human clone which has transferred to a dairy cow and has subsequently caused mastitis. The detection of indistinguishable *S. aureus* isolates from bovine and human hosts at three of the sampling sites is suggestive of bacterial transmission and supports the need for vigilant monitoring of staphylococcal populations at the human-animal interface.

## Introduction

*Staphylococcus aureus* is a versatile pathogen responsible for a variety of infections in humans and animals (Hata et al., 2008). In humans *S. aureus* is responsible for a number of conditions ranging from superficial skin infections to life-threatening diseases, such as endocarditis and hemolytic pneumonia (Lindsay and Holden, 2004). Additionally, through the production of toxins, *S. aureus* can cause specific toxin-mediated conditions, such as scalded skin syndrome, staphylococcal food poisoning and toxic shock syndrome (Becker et al., 2015). In animals, *S. aureus* is a common etiological agent of mastitis, an infectious disease condition responsible for significant financial losses to dairy farmers worldwide (Petrovski et al., 2006; Fitzgerald, 2012).

The success of *S. aureus* as a pathogen is attributable, in part, to the diverse range of virulence factors produced (Gordon and Lowy, 2008). The virulence factors facilitate the invasion and colonization of host tissue, evasion of the hosts' immune defence mechanisms, aid in acquisition of nutrients and dissemination of the bacteria within the host tissue (Ferry et al., 2005; Haveri et al., 2008). Among the vast array of virulence factors produced are numerous enzymes and cytotoxins, such as coagulase, collagenase, exfoliative toxins, hemolysins, hyaluronidase, leukocidins, lipases, nucleases and staphylokinase (Smeltzer et al., 2009). Most *S. aureus* strains are able to produce one or more pyrogenic toxin superantigens (PTSAgs) which includes staphylococcal enterotoxins and the structurally related protein, toxic shock syndrome toxin-1 (TSST-1) (Wright and Novick, 2003). All of these toxins exhibit superantigenic activity by interacting with antigen-presenting cells and T-lymphocytes irrespective of the antigen specificity of the cells (Akineden et al., 2001; Argudín et al., 2010). This interaction leads to a massive proliferation of T-cells and the uncontrolled release of pro-inflammatory cytokines that can lead to clinical signs in the host that include, fever, hypotension and shock (Plata et al., 2009; Argudín et al., 2010). The production of enterotoxins is particularly significant from a public health standpoint as the ingestion of preformed toxins is a major cause of food poisoning worldwide (Le Loir et al., 2003; Srinivasan et al., 2006). The expression of most staphylococcal virulence factors occurs in a co-ordinated manner and is strictly controlled by a series of regulatory genes which operate under the control of the accessory gene regulator (*agr*) (Ben Ayed et al., 2008; Lowy, 2013).

*Staphylococcus aureus* is a contagious mastitis pathogen with the milk from infected mammary glands serving as the primary reservoir of the bacterium which may be transferred to other animals in the herd during milking (Capurro et al., 2010). Several epidemiological studies have suggested that a few specialized *S. aureus* strains are responsible for the majority of bovine intramammary infections (IMIs) worldwide (Kapur et al., 1995; Zadoks et al., 2002; Sakwinska et al., 2011; Budd et al., 2015). However, the occurrence of herd-specific strains has also been reported (Joo et al., 2001; Sommerhäuser et al., 2003; Rabello et al., 2007; Oliveira et al., 2011). Moreover, it has been demonstrated that different strains are associated with different clinical outcomes in the host including severity and persistence of infections and response to antimicrobial treatment (Haveri et al., 2005, 2007; Lundberg et al., 2014). A better understanding of the epidemiology, including transmission and virulence characteristics of *S. aureus* strains may assist with the development of strategies to control the spread of the bacterium within herds (Haveri et al., 2005, 2008).

In the dairy environment documented evidence of methicillin-resistant *S. aureus* transmission between cows and close human contacts exists (Juhász-Kaszanyitzky et al., 2007; Haenni et al., 2010). Further, reports of livestock-associated MRSA (LA-MRSA) ST398 causing mastitis have created additional public health concerns regarding production animals, including dairy cows, as a source of antimicrobial-resistant bacteria which can spill-over into humans (Monecke et al., 2007; Feßler et al., 2010; Fitzgerald, 2012; Tenhagen et al., 2014). It is now commonly accepted that persons working in close contact with animals are at a higher risk of being colonized or even infected with zoonotic bacteria carried by animals than individuals who do not interact with animals (Graveland et al., 2011). It is therefore recommended that surveillance at the interface between human and animal hosts is monitored in order to detect any potential, emerging human health risks (Fitzgerald, 2012; Cuny et al., 2015).

A number of genotyping methods have been used to characterize *S. aureus* isolates including virulence gene profiling, *agr* typing, pulsed-field gel electrophoresis (PFGE), *spa* sequence typing and multilocus sequence typing (MLST) (Hata et al., 2010; Kadlec et al., 2015). The application of whole genome sequencing (WGS) has gained traction during the past decade as technological advances have improved the accessibility and cost-effectiveness of high-throughput sequencing (Trees et al., 2015). At present however, WGS still remains unaffordable in resource-limited settings.

In a previous study our group investigated the diversity and antimicrobial susceptibility of staphylococci responsible for causing bovine IMIs (Schmidt et al., 2015). Concurrent sampling and characterization of staphylococci from close contact workers facilitated a comparison between isolates from different hosts. To date no genotyping studies have been undertaken in South Africa to investigate the genetic diversity of *S. aureus* strains causing IMIs and the potential public health risk posed by these bacterial populations. Using our previous work as a platform the purpose of the present study was to (i) screen isolates for clinically significant antimicrobial and virulence gene markers including: antimicrobial resistance (*bla*Z, *mec*A, *mec*C, *van*A, *van*B), exfoliative toxin (*eta, etb, etd*), hemolysin (*hla, hlb, hld, hlg, hlg2*), leukocidin (*luk*ED, *luk*M, *luk*S/F) and pyrogenic superantigen toxins (*sea* to *see, seg* to *sei, selj* to *selr, selu* and *tst*) among bovine and human *S. aureus* isolates; (ii) investigate the diversity of *S. aureus* genotypes causing mastitis in dairy herds through genomic macrorestriction and PFGE analysis of isolates; (iii) relate the occurrence of local genotypes to global *S. aureus* lineages, and (iv) evaluate the genetic relatedness of *S. aureus* isolates causing mastitis and isolates recovered from humans working in close contact with the animals.

## Materials and methods

### Ethics statement

Ethical clearance for this investigation was obtained from the Animal Ethics Committee (Faculty of Veterinary Science, H010-13) and the Research Ethics Committee (Faculty of Health Sciences, 295/2013) of the University of Pretoria. Participation in this study was voluntary and written informed consent was obtained from all animal owners and human contacts prior to sampling.

### Bacterial isolates

**Figure 1:**
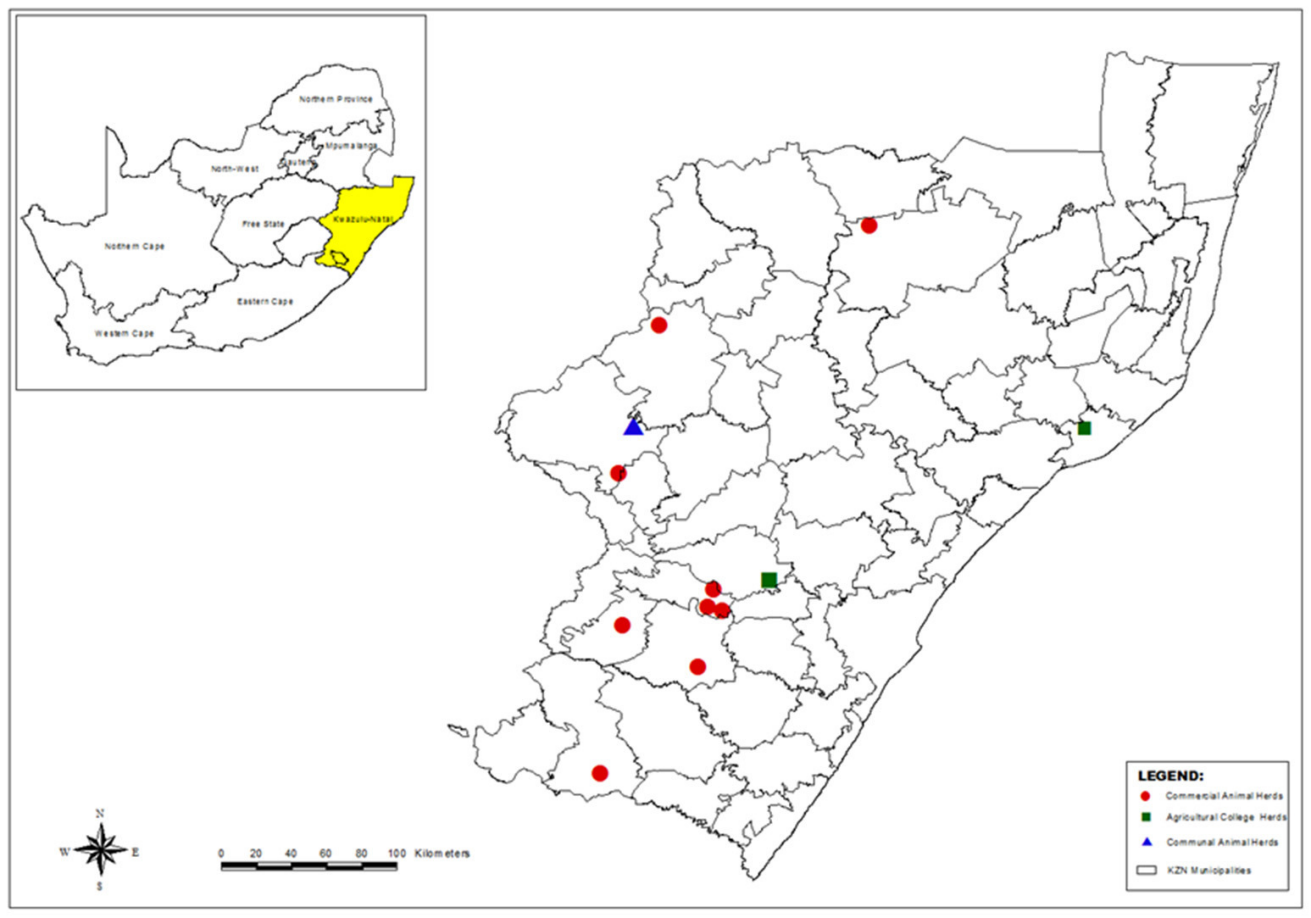
**Map of KwaZulu-Natal, South Africa, showing the municipal districts and the location of the dairy herds where sampling was carried out (Generated using ArcMap version 10)**.

A total of 146 *S. aureus* isolates from milk and 12 isolates from human nasal swabs were characterized in this study. The samples originated from 13 sampling sites (identified by letters A to J and L to M) located in the province of KwaZulu-Natal (KZN), South Africa (Figure 1). At the time of sampling animal owners were asked to provide details regarding the: average size of their dairy herd; intramammary preparations used; number of laborers working in the dairy; and the source of new heifers.

Sample collection, processing and bacteriological analyses are described in detail elsewhere (Schmidt et al., 2015). All *Staphylococcus aureus* isolates were identified by means of standard phenotyping methods including coagulase production using rabbit plasma (Bio-Rad, USA) and the detection of clumping factor, fibronectin-binding proteins and protein A using the Pastorex™ StaphPlus latex agglutination test kit (BioRad, USA). Phenotypic results were confirmed using a multiplex-PCR (M-PCR) assay (Schmidt et al., 2015).

### Detection of virulence factors and immune evasion cluster genes

The genomic DNA of all test isolates was extracted using the DNeasy blood and tissue kit (Qiagen, Germany) in accordance with the manufacturer's instructions. Isolates were evaluated using nine M-PCR assays (AMR1, VGP1 to VGP8) to screen for antimicrobial resistance (*bla*Z, *mec*C, *van*A, and *van*B), exfoliative (*eta, etb, etd*), hemolysin (*hla, hlb, hld, hlg, hlg*2), leukocidin (*luk*M, *luk*ED) and PTSAg (*sea* to *see, seg* to *sei, selj* to *selr, seu, tst*) genes. Multiplex PCR assays were carried out using the Qiagen Multiplex PCR kit (Germany) according to the manufacturer's instructions. The presence of the human immune evasion cluster (IEC) genes, *chp, sak* and *scn* were assayed using separate uniplex PCR assays. Two microliters of each DNA extract was added to PCR reaction mixtures comprising of 1X PCR buffer (Promega, USA), 1.5 mM MgCl_2_(Promega, USA), 0.4 mM dNTPs (Promega, USA), 0.5 μM of each primer and 2.5 IU HotStart Taq (Promega, USA).

Details of all the oligonucleotide primers, PCR assay conditions and positive control isolates used are summarized in Table 1. All PCR reactions were cycled in a MJ Mini thermocycler (BioRad, USA) with appropriate positive (Table 1) and negative (deionized water) controls. Amplification products were electrophoresed in a 1.8% (m/v) agarose gel (SeaKem, Lonza, USA) in a 1X Tris-borate-EDTA (TBE) buffer (Melford, UK). Following staining in a 1 μg/mL ethidium bromide solution (10 mg/mL) (BioRad, USA) the PCR products were visualized and documented (UVIPro, Uvitec, UK). A 100 bp molecular weight marker (MWM) (Solis BioDyne, Estonia or Thermo Scientific, USA) was electrophoresed alongside samples to enable the size of the PCR products to be determined.

**Table 1:** **Oligonucleotide primer sequences and details for the PCR-based assays used in this study to characterize ***S.aureus*****.

**Gene**	**Primer name**	**Oligonucleotide sequence (5′–3′)^†^**	**Size of PCR amplicon (bp)**	**Positive control**	**References**
16S rRNA^*^	Staph 756F	AACTCTGTTATTAGGGAAGAACA	756	None	McClure et al., 2006
	Staph 750R	CCACCTTCCTCCGGTTTGTCACC			
**AMR1^a^**					
*bla*Z	blaZ-F	AAGAGATTTGCCTATGCTTC	517	*S. aureus*	Vesterholm-Nielsen et al., 1999
	blaZ-R	GCTTGACCACTTTTATCAGC		ATCC 29213	
*mec*C	mecA_LGA251_ MultiFP	GAAAAAAAGGCTTAGAACGCCTC	138	*S. aureus* field isolate 13ANR24389_RV	Stegger et al., 2012
	mecA_LGA251_ MultiRP	GAAGATCTTTTCCGTTTTCAGC			
*van*A	vanA-F	CATGAATAGAATAAAAGTTGCAATA	1,030	*Enterococcus faecalis*	Clark et al., 1993
	vanA-R	CCCCTTTAACGCTAATACGATCAA		ATCC 70021	
*van*B	vanB-F	GTGACAAACCGGAGCGAGGA	433	*E. faecalis*	
	vanB-R	CCGCCATCCTCCTGCAAAAAA		ATCC 51299	
**VGP1^a^**					
*sea*	GSEAR-1	GGTTATCAATGTGCGGGTGG	102	*S. aureus* field isolate 00V08510	Mehrotra et al., 2000
	GSEAR-2	CGGCACTTTTTTCTCTTCGG			
*seb*	GSEBR-1	GTATGGTGGTGTAACTGAGC	164	*S. aureus* field isolate 00V08510	
	GSEBR-2	CCAAATAGTGACGAGTTAGG			
*sec*	GSECR-1	AGATGAAGTAGTTGATGTGTATGG	451	*S. aureus* field isolate	
	GSECR-2	CACACTTTTAGAATCAACCG		13ANR24389_RV	
*sed*	GSEDR-1	CCAATAATAGGAGAAAATAAAAG	278	*S. aureus* field isolate	
	GSEDR-2	ATTGGTATTTTTTTTCGTTC		13ANR12816	
*see*	GSEER-1	AGGTTTTTTCACAGGTCATCC	209	*S. aureus* field isolate	
	GSEER-2	CTTTTTTTTCTTCGGTCAATC		Jena_FLI W56	
**VGP2^a^**					
*seg*	SEG-1	AAGTAGACATTTTTGGCGTTCC	287	*S. aureus* field isolate	Omoe et al., 2002, 2005
	SEG-2	AGAACCATCAAACTCGTATAGC		13ANR12816	
*seh*	SEH-1	GTCTATATGGAGGTACAACACT	213	*S. aureus* field isolate	
	SEH-2	GACCTTTACTTATTTCGCTGTC		05-Student-18	
*sei*	SEI-1	GGTGATATTGGTGTAGGTAAC	454	*S. aureus* field isolate	
	SEI-2	ATCCATATTCTTTGCCTTTACCAG		13ANR12816	
*selj*	SEJ-1	ATAGCATCAGAACTGTTGTTCCG	152	*S. aureus* field isolate	
	SEJ-2	CTTTCTGAATTTTACCACCAAAGG		13ANR12816	
*selp*	SEP-3	TGATTTATTAGTAGACCTTGG	396	*S. aureus* field isolate	
	SEP-4	ATAACCAACCGAATCACCAG		13ANR12816	
**VGP3^a^**					
*selk*	SEK-1	TAGGTGTCTCTAATAATGCCA	293	*S. aureus* field isolate 00V08510	Omoe et al., 2005
	SEK-2	TAGATATTCGTTAGTAGCTG			
*selm*	SEM-1	GGATAATTCGACAGTAACAG	379	*S. aureus* field isolate	
	SEM-2	TCCTGCATTAAATCCAGAAC		13ANR12816	
*selo*	SEO-1	TGTGTAAGAAGTCAAGTGTAG	214	*S. aureus* field isolate	
	SEO-2	TCTTTAGAAATCGCTGATGA		13ANR12816	
*tst1*	GTSSTR-1	ACCCCTGTTCCCTTATCATC	326	*S. aureus* field isolate	Mehrotra et al., 2000
	GTSSTR-2	TTTTCAGTATTTGTAACGCC		05-Student-18	
**VGP4^a^**					
*sell*	SEL-1	TAACGGCGATGTAGGTCCAGG	383	*S. aureus* field isolate	Omoe et al., 2005
	SEL-2	CATCTATTTCTTGTGCGGTAAC		07V33069	
*selq*	SEQ-1	AATCTCTGGGTCAATGGTAAGC	122	*S. aureus* field isolate 00V08510	
	SEQ-2	TTGTATTCGTTTTGTAGGTATTTTCG			
*selr*	SER-1	GGATAAAGCGGTAATAGCAG	166	*S. aureus* field isolate	
	SER-4	GTATTCCAAACACATCTAAC		13ANR12816	
**VGP5^b^**					
*seln*	Mp-sen-1	ATGAGATTGTTCTACATAGCTGCAAT	680	*S. aureus* field isolate	Jarraud et al., 2002
	Mp-sen-2	AACTCTGCTCCCACTGAAC		13ANR12816	
*selu*	SEU-F	ATCAGAAACAAACATTAAAGCCA	500	*S. aureus* field isolate	Park et al., 2011
	SEU-R	TGACCATTTCCTTCGATAAACTTTAT		13ANR12816	
**VGP6^b^**					
*hla*	HLA-1	CTGATTACTATCCAAGAAATTCGATTG	209	*S. aureus* field isolate	Jarraud et al., 2002
	HLA-2	CTTTCCAGCCTACTTTTTTATCAGT		05-Student-18	
*hlb*	HLB-1	GTGCACTTACTGACAATAGTGC	309	*S. aureus* field isolate	
	HLB-2-2	GTTGATGAGTAGCTACCTTCAGT		11ANR780898	
*hld*	HLD-1	AAGAATTTTTATCTTAATTAAGGAAGGAGTG	111	*S. aureus* field isolate	
	HLD-2	TTAGTGAATTTGTTCACTGTGTCGA		05-Student-18	
*hlg*	mpHLG-1	GTCAYAGAGTCCATAATGCATTTAA	535	*S. aureus* verified field isolate UP-MR40	
	mpHLG-2	CACCAAATGTATAGCCTAAAGTG			
*hlg*2	mpHLG2-1	GACATAGAGTCCATAATGCATTYGT^d^	390	*S. aureus* verified field isolate UP-MR42	
	mpHLG2-2	ATAGTCATTAGGATTAGGTTTCACAAAG			
**VGP7^a^**					
*eta*	GETAR-1	GCAGGTGTTGATTTAGCATT	93	*S. aureus* field isolate	Mehrotra et al., 2000
	GETAR-2	AGATGTCCCTATTTTTGCTG		07V33069	
*etb*	GETBR-1	ACAAGCAAAAGAATACAGCG	226	*S. aureus* field isolate	
	GETBR-2	GTTTTTGGCTGCTTCTCTTG		07V33069	
*etd*	ET-14	AACTATCATGTATCAAGG	376	*S. aureus* field isolate	Yamaguchi et al., 2002
	ET-15	CAGAATTTCCCGACTCAG		04V16073	
**VGP8^b^**					
*luk*M	lukM-1	TGGATGTTACCTATGCAACCTAC	780	*S. aureus* field isolate	Jarraud et al., 2002
	lukM-2	GTTCGTTTCCATATAATGAATCACTAC		RF122	
*luk*ED	lukDE-1	TGAAAAAGGTTCAAAGTTGATACGAG	269	*S. aureus* field isolate	
	lukDE-2	TGTATTCGATAGCAAAAGCAGTGCA		RF122	
**UNIPLEX ASSAYS^c^**					
*chp*		TTTTTAACGGCAGGAATCAGTA	404	*S. aureus* verified field isolate UP-MR40	Sung et al., 2008
		TGCATATTCATTAGTTTTTCCAGG			
*sak*		TGAGGTAAGTGCATCAAGTTCA	403	*S. aureus* field isolate 00V08510	
		CCTTTGTAATTAAGTTGAATC CAGG			
*scn*		ATACTTGCGGGAACTTTAGCAA	320	None	
		TTTTAGTGCTTCGTCAATTTCG			
**ACCESSORY GENE REGULATOR TYPING^c^**					
*agr*	agr1	GTCACAAGTACTATAAGCTGCGAT	441	*S. aureus*	Gilot et al., 2002 Smyth et al., 2009
	PAN	ATGCACATGGTCGACATGC		ATCC 16600	
	Agr2	TATTACTAATTGAAAAGTGGCCATAGC	575	*S. aureus*	
	PAN	ATGCACATGGTCGACATGC		ATCC 29213	
	Agr3	GTAATGTAATAGCTTGTATAATAATACCCAG	323	*S. aureus*	
	PAN	ATGCACATGGTCGACATGC		ATCC 25923	
	Agr4	CGATAATGCCGTAATACCCG	659	*S. aureus* field isolate	
	PAN	ATGCACATGGTCGACATGC		07V33069	
**SEQUENCE TYPING OF THE STAPHYLOCOCCAL PROTEIN A GENE^c^**					
*spa*	1095F	AGACGATCCTTCGGTGAGC	Variable	None	Shopsin et al., 1999
	1517R	GCTTTTGCAATGTCATTTACTG			Harmsen et al., 2003
**MULTILOCUS SEQUENCE TYPING^c^**					
*arc*C	*arc*-Up	TTGATTCACCAGCGCGTATTGTC	456	None	Enright et al., 2000
	*arc*-Dn	AGGTATCTGCTTCAATCAGCG			
*aro*E	*aro*E-Up	ATCGGAAATCCTATTTCACATTC	456	None	
	*aro*E-Dn	GGTGTTGTATTAATAACGATATC			
*glp*F	*glp*F-Up	CTAGGAACTGCAATCTTAATCC	465	None	
	*glp*F-Dn	TGGTAAAATCGCATGTCCAATTC			
*gmk*	*gmk*-Up	ATCGTTTTATCGGGACCATC	429	None	
	*gmk*-Dn	TCATTAACTACAACGTAATCGTA			
*pta*	*pta*-Up	GTTAAAATCGTATTACCTGAAGG	474	None	
	*pta*-Dn	GACCCTTTTGTTGAAAAGCTTAA			
*tpi*	*tpi*-Up	TCGTTCATTCTGAACGTCGTGAA	402	None	
	*tpi*-Dn	TTTGCACCTTCTAACAATTGTAC			
*yqi*L	*yqi*L-Up	CAGCATACAGGACACCTATTGGC	516	None	
	*yqi*L-Dn	CGTTGAGGAATCGATACTGGAAC			

† *All oligonucleotides were synthesized and purified by Inqaba Biotechnical Industries, South Africa with the exception of GSEAR-1, GSEAR-2, GSEBR-1, GSEBR2, GSECR-1, GSECR-2, GSEDR-1, GSEDR-2, GSEER-1, GSEER-2, GETAR-1, GETAR-2, GETBR-1, GETBR-2, GTSSTR-1 GTSSTR-2, lukDE-1, lukDE-2, LukM-1, and lukM-2, which were synthesized by Integrated DNA Technologies (IDT®) (California, USA)*.

* *The 16S primer pair was included in the following M-PCR assays: AMR1, VGP1, VGP2, VGP3, VGP4, VGP 6, and VGP7*.

a *Annealing temperature 57°C*.

b *Annealing temperature 51°C*.

c *Annealing temperature 55°C*.

d *Y = T or C*.

### Accessory gene regulator (*agr*) typing

The *agr* allele types (I-IV) of all *S. aureus* isolates were determined as described by Gilot et al. (2002). The multiplex-PCR assay comprised of a forward primer, Pan, common to all *agr* groups and four reverse primers each one specific to each *agr* group (Gilot et al., 2002). Amplification products were electrophoresed as described in Section Detection of Virulence Factors and Immune Evasion Cluster Genes.

### Genomic macrorestriction and pulsed-field gel electrophoresis of *Staphylococcus aureus* isolates

All *S. aureus* isolates were characterized by macrorestriction of genomic DNA using *Sma*I followed by PFGE of the restriction products. The procedure followed is based on protocols described by Graves and Swaminathan (2001) and McDougal et al. (2003) but with several modifications. *Staphylococcus aureus* ATCC 12600 was processed and electrophoresed together with each batch of test samples. Bacterial cell suspensions were prepared in TE buffer (10 mM Tris-HCL, 1 mM EDTA, pH 8.0, Sigma-Aldrich, USA) and the turbidity adjusted to 1.2–1.6 using a microplate reader at 630 nm (ELx 800, BioTEK, USA). Twenty microliters of lysozyme (20 mg/mL; Sigma-Aldrich, USA) was added to 400 μL of each cell suspension and incubated (LabNet International Inc., USA) at 56°C for 20 min. Following incubation, 20 μL proteinase K (20 mg/mL; Roche, Germany) and 5 μL lysostaphin (1 mg/mL; Sigma-Aldrich, USA) was added to each sample before an equal volume of molten 1.2% (m/v) SeaKem® LE agarose (Lonza, USA) was added. Each sample suspension was gently mixed before being transferred to a re-usable plug mold (Biometra, Germany) and allowed to solidify. Sample plugs were transferred to separate 15 mL conical tubes (Falcon, México) containing 5 mL cell lysis buffer (50 mM Tris-HCl pH 8.0, 50 mM EDTA, pH 8.0, 1% sodium lauryl sarcosine, Sigma-Aldrich, USA) and 25 μL proteinase K (20 mg/mL) (Roche, Germany). Sample tubes were incubated (Stuart®, Bibby Scientific Ltd., UK) for 18–24 h at 51°C with constant agitation at 170 rpm. Following incubation the plugs were washed as described by Graves and Swaminathan (2001). Appropriately sized slices were cut from each plug and digested with 50 IU *Sma*I (New England BioLabs, USA) at 37°C for 2 h. Plug slices were sealed in the wells of a 1.2% (m/v) SeaKem® LE agarose gel (Lonza, USA) and electrophoresed in 0.25X TBE buffer (pH 8.3; Sigma-Aldrich, USA) using the Rotaphor® system (Biometra, Germany). The electrophoretic parameters were set as follows: switch times of 5–40 s; angle 120° constant; 220 V linear to 200 V for 25 h at 13°C. Following electrophoresis the gel was stained in a 0.25 μg/mL ethidium bromide solution (10 mg/mL; Sigma-Aldrich, USA) for 15 min and de-stained in deionized water for 30 min. Gels were visualized and captured using a gel documentation system (UVPro, Uvitec, UK). All gel images were analyzed using the GelCompar *II* software program (Applied Maths, Belgium). Similarity co-efficients were calculated and a dendrogram constructed using the Dice coefficient and UPGMA. A band position tolerance of 1.0% was used and the cluster (pulsotype, PT) cut-off was set at an 80% similarity level which corresponds to the Tenover et al. (1995) criteria of four to six band differences between related isolates (McDougal et al., 2003; Faria et al., 2008). All pulsotypes were assigned Arabic numerals and were classified as being either major pulsotypes (≥5 isolates), minor pulsotypes (two to three isolates) or singletons. Sixteen bovine isolates, representative of all pulsotypes, and 10 isolates of human origin were genotyped further using sequence analysis of the *spa* gene and MLST.

### Sequence typing of the staphylococcal protein A gene of *Staphylococcus aureus* isolates

The polymorphic X-Region of the *spa* gene was amplified according to the protocol of Harmsen et al. (2003) and the PCR amplicons were submitted to Inqaba Biotechnical Industries (South Africa) for sequencing. Sequence files were assembled using CLC Main Workbench Version 6.0 (CLCbio, USA) before being imported and analyzed in DNAgear (201203012225). Novel repeat sequence profiles and trace data files were submitted to the Ridom Spa server database curator for evaluation and assignment of new *spa* types.

### Multilocus sequence typing analysis of *Staphylococcus aureus* isolates

The internal fragments of seven *S. aureus* housekeeping genes (*arc*C, *aro*E, *glp*F, *gmk, pta, tpi*, and *yqi*L) were amplified as previously described (Enright et al., 2000). The PCR amplicons were submitted to Inqaba Biotechnical Industries (South Africa) for DNA sequencing of both the forward and reverse strands. Following assembly of the DNA sequences in CLC Main Workbench version 6.0 (CLCbio, USA) the sequence of each locus was compared to allele sequences in the MLST database (http://saureus.mlst.net). The combination of alleles at the seven loci was used to define the allelic profile for each isolate and assigned to a sequence type (ST). The eBURST algorithm (version 3.0; last previous update 24/9/2015) was used to assign MLST clonal complexes, with the minimum number of common alleles set at six of seven (Feil et al., 2003). Trace files of putative novel alleles and the allelic profiles of novel STs were submitted to the curator of the database for evaluation, assignment of allele or ST number and entry into the database.

## Results

The distribution of samples and *S. aureus* isolates recovered at each of the sampling sites is summarized in Table 2. For further details pertaining to sampling and selection of bacterial isolates the reader is referred to Schmidt et al. (2015).

**Table 2:** **Distribution of ***Staphylococcus aureus*** isolates and pulsotypes from bovine intramammary infections and human nasal swabs collected from 13 sampling sites in the province of KwaZulu-Natal, South Africa**.

**Sampling sites**	**Average size of milking herd**	**Bovine milk samples**		**Human nasal swab specimens**	
		**Percentage of sampled cows positive for *S.aureus***	Pulsotypes^*^ (number of isolates)	**Percentage of specimens positive for *S.aureus***	Pulsotypes^*^ (number of isolates)
**COMMERCIAL DAIRY HERDS (*****n*** = **9)**					
A	400	5.6 (5/89)	PT7 (5)	12.5 (1/8)	PT14 (1)
B	700	25.9 (14/54)	PT7 (12), PT8 (2)	25 (2/8)	PT17 (2)
C	300	4.8 (3/63)	PT7 (3)	7.1 (1/14)	PT12 (1)
E	1,100	10.5 (29/277)	**PT2** (29)	12.5 (1/8)	**PT2** (1)
F	500	18.3 (22/120)	PT7 (12), PT10 (10)	14.3 (1/7)	PT13 (1)
G	95	14.5 (18/124)	PT1 (9), PT2 (7), PT7 (1), PT8 (1)	0 (0/4)	–
I	500	21.4 (21/98)	PT1 (12), PT3 (1), PT4 (3), PT6 (1), PT7 (4)	0 (0/6)	–
J	600	0 (0/281)	–	27.3 (3/11)	PT11 (2), PT15 (1)
L	1,450	7.8 (16/206)	PT5 (1), PT7 (14), **PT9** (1)	14.3 (1/7)	**PT9** (1)
**AGRICULTURE COLLEGE DAIRY HERDS (*****n*** = **2)**					
D	40	39.5 (15/38)	**PT7** (15)	50 (1/2)	**PT7** (1)
H	50	37.5 (3/8)	PT7 (3)	0 (0/2)	–
**COMMUNAL ANIMAL HERDS (*****n*** = **2)**					
M	Unknown	0 (0/16)	–	50 (1/2)	PT16 (1)

* *Pulsotypes common to both bovine and human S. aureus are shown in bold*.

### Phenotypic and molecular identification of *Staphylococcus aureus* isolates

All *S. aureus* isolates tested positive for coagulase production using rabbit plasma. With the exception of 10 isolates from sampling site F, all *S. aureus* isolates tested positive with the Pastorex™ StaphPlus agglutination test kit. The phenotypic identification of *S. aureus* isolates was confirmed using the species-specific multiplex-PCR assay.

### Virulence gene profiling of *Staphylococcus aureus* isolates

The distribution of virulence genes between the different *S. aureus* pulsotypes is shown in Table 3. None of the bovine or human *S. aureus* isolates tested positive for the presence of either of the methicillin resistance genes, *mec*A or *mec*C, or the vancomycin resistance genes, *van*A or *van*B. The *bla*Z gene was detected in 28.8% (42/146) and 75% (8/12) of the bovine and human *S. aureus* isolates respectively. All *S. aureus* isolates tested positive for the hemolysin genes, *hla* and *hld*. The *hlg* and *hlg2* genes were detected in all the bovine isolates and 75% (9/12) and 67% (8/12) of the human isolates respectively. The β-hemolysin gene was detected in 86% (126/146) of the bovine isolates and only 25% (3/12) of the human isolates. None of the *S. aureus* isolates tested positive for the Panton-Valentine leukocidin toxin gene, *luk*S/F. All bovine *S. aureus* isolates tested positive for *luk*ED whilst this leukocidin gene was found in only 75% (8/12) of the isolates of human origin. Comparatively, the *luk*M gene was detected in 56.2% (82/146) of the bovine isolates and only a single human isolate. None of the bovine isolates tested positive for the exfoliative toxin genes *eta, etb*, or *etd*. Only a single human *S. aureus* isolate tested positive for the *eta* gene.

**Table 3:** **Genotypic characteristics of ***S. aureus*** isolates from bovine intramammary infections and close human contacts**.

**PT**	**Origin of isolates**	**Sampling site (no. of isolates)**	*Spa* type^*^	ST^*^ (CC)	***agr* type**	**Antimicrobial resistance genes**	**Immune evasion cluster genes^†^**	**Cytotoxin genes detected^†^**	**PTSAg genes detected^†^**
PT1 (major)	Bovine	G (9)	**t15533**	ST2992 (CC97)	I	*bla*Z	*chp^1^, sak*	*hla, hlb, hld, hlg, hlg*2, *luk*ED	
		I (12)	**t15539**	ST2992 (CC97)	I	*bla*Z		*hla, hlb*^1^, *hld, hlg, hlg*2, *luk*ED	
PT2 (major)	Bovine	E (29)	t189	ST97 (CC97)	I	Negative		*hla, hlb, hld, hlg, hlg*2, *luk*ED	*sec*^28^, *sell*^28^
		G (7)			I	Negative		*hla, hlb, hld, hlg, hlg*2, *luk*ED	
	Human	E (1)	t189	ST97 (CC97)	I	Negative		*hla, hlb, hld, hlg, hlg*2, *luk*ED	*sec, sell*
PT3	Bovine	I (1)	**t15539**	ST2992 (CC97)	I	*bla*Z	*sak*	*hla, hld, hlg, hlg*2, *luk*ED	
PT4 (minor)	Bovine	I (3)	**t15539**	ST2992 (CC97)	I	*bla*Z	*sak*	*hla, hld, hlg, hlg*2, *luk*ED	
PT5	Bovine	L (1)	**t15536**	**ST3538** (CC97)	I	*bla*Z	*sak*	*hla, hld, hlg, hlg*2, *luk*ED	
PT6	Bovine	I (1)	**t15539**	ST2992 (CC97)	I	*bla*Z	*sak*	*hla, hld, hlg, hlg*2, *luk*ED	
PT7 (major)	Bovine	A (5)	**t15538**	ST352 (CC97)	I	Negative		*hla, hlb, hld, hlg, hlg*2, *luk*ED, *luk*M	
		B (12)			I	*bla*Z		*hla, hlb, hld, hlg, hlg*2, *luk*ED, *luk*M	
		C (3)	t730	ST352 (CC97)	I	Negative		*hla, hlb, hld, hlg, hlg*2, *luk*ED, *luk*M	
		D (15)	t1201	ST352 (CC97)	I	Negative		*hla, hlb, hld, hlg, hlg*2, *luk*ED, *luk*M	
		F (12)	t1201	ST352 (CC97)	I	Negative		*hla, hlb, hld, hlg, hlg*2, *luk*ED, *luk*M	
		G (1)			I	Negative		*hla, hlb, hld, hlg, hlg*2, *luk*ED, *luk*M	
		H (3)	t1201	ST352 (CC97)	I	Negative		*hla, hlb, hld, hlg, hlg*2, *luk*ED, *luk*M	
		I (4)			I	Negative		*hla, hlb*^3^, *hld, hlg, hlg*2, *luk*ED, *luk*M	
		L (14)			I	Negative		*hla, hlb*^13^, *hld, hlg, hlg*2, *luk*ED, *luk*M	
	Human	D(1)	t1201	ST352 (CC97)	I	Negative		*hla, hlb, hld, hlg, hlg*2, *luk*ED, *luk*M	
PT8 (minor)	Bovine	B (2)	t693	ST352 (CC97)	I	*bla*Z		*hla, hlb, hld, hlg, hlg*2, *luk*ED, *luk*M	
		G (1)	t015	ST508 (CC45)	I	Negative		*hla, hlb, hld, hlg, hlg*2, *luk*ED, *luk*M	
PT9	Bovine	L (1)	t064	ST8 (CC8)	I	*bla*Z	*sak*	*hla, hld, hlg, hlg*2, *luk*ED	*sea*
	Human	L (1)	t064	ST8 (CC8)	I	*bla*Z	*sak*	*hla, hld, hlg, hlg*2, *luk*ED	*sea*
PT10 (major)	Bovine	F (10)	t529	ST351 (CC705)	II	Negative		*hla, hlb, hld, hlg, hlg*2, *luk*ED, *luk*M	*sec, seg, seli, sell, seln, selu, tst*
PT11 (minor)	Human	J (2)	t1476	ST8 (CC8)	I	*bla*Z	*chp, sak*	*hla, hld, hlg, hlg*2, *luk*ED	*selj, selr*
PT12	Human	C (1)	t360	ST15 (CC15)	II	*bla*Z	*chp*	*eta, hla, hld, hlg, hlg*2, *luk*ED	
PT13	Human	F (1)	t581	ST5 (CC5)	II	*bla*Z		*hla, hlb, hld, hlg, hlg*2, *luk*ED	*sed, seg, sei, selj selm seln, selo, selr, seu*
PT14	Human	A (1)	t2172	ST707 (CC707)	III	*bla*Z	*chp, sak*	*hla, hld, hlg, hlg*2, *luk*ED	*selk, selq, tst*
PT15	Human	J (1)	t7023	ST45 (CC45)	I	*bla*Z	*chp, sak*	*hla, hld, hlg*	*sec, selg, seli, sell, selm, seln, selo, selu*
PT16	Human	M (1)	t015	ST508 (CC45)	I	Negative	*chp, sak*	*hla, hld*	*sec, seg, seli, sell, selm, selo, tst*
PT17 (minor)	Human	B (2)	t012	ST30 (CC30)	III	*bla*Z	*sak*	*hla, hld*	*seg*

* New spa types and sequence type are shown in bold font;

† *In some cases not all of the isolates in the group presented with the indicated genes. Where this occurred the number of isolates positive for the gene is indicated by a superscript; CC, Clonal complex; PT, Pulsotype; PTSAg, pyrogenic toxin superantigen; ST, sequence type*.

Overall 27% (39/146) of the bovine *S. aureus* isolates and 83.3% (10/12) of the *S. aureus* isolates of human origin tested positive for one or more PTSAg genes. The PTSAg gene positive bovine *S. aureus* were isolated from only three of the sampling sites namely, commercial dairy herds E, F and L. The *sec* and *sell* genes were the most common genes detected among the bovine isolates with the *sec*-*sell* gene combination occurring in 71.8% (28/39) of the PTSAg-positive *S. aureus* isolates. Comparatively, a more diverse range of PTSAg toxin genes were detected among the human isolates including: *sea* (*n* = 1), *sec* (*n* = 3), sed (*n* = 1), *seg* (*n* = 5), sei (*n* = 3), *selj* (*n* = 3), *selk* (*n* = 1), *sell* (*n* = 3), *selm* (*n* = 3), *seln* (*n* = 2), *selo* (*n* = 3), *selq* (*n* = 1), *selr* (*n* = 3), *selu* (*n* = 2), and *tsst*-1 (*n* = 2). None of the bovine or human *S. aureus* isolates tested positive for the *seb, sed, see, seh*, or *selp* genes. The immune evasion cluster genes, *chp* and *sak*, which code the chemotaxis inhibitory protein of *S. aureus* (CHIPS) and the staphylokinase (SAK) enzyme, were detected in 0.7% (1/146) and 13% (19/146) of the bovine *S. aureus* isolates evaluated. Among the human isolates the *chp* and *sak* genes were detected in 50% (6/12) and 66.7% (8/12) of the isolates evaluated. None of the isolates carried the *scn* gene encoding the staphylococcal complement inhibitor protein (SCIN).

### Accessory gene regulator (*agr*) typing

The *agr* of all *S. aureus* isolates could be characterized using the described *agr* M-PCR assay of Gilot et al. (2002). Among the isolates of bovine origin *agr* type I was the dominant type observed, with 93.2% (136/146) of the isolates found to be of this type. The remaining 6.8% (10/146) of the bovine isolates were *agr* type II. Among the 12 *S. aureus* isolates recovered from the human nasal swabs, 58.3% (7/12), 16.7% (2/12), and 25% (3/12) were identified as *agr* types I, II and III respectively (Table 3). No *S. aureus* isolates were found to have a type IV accessory gene regulator.

### Genomic macrorestriction and pulsed-field gel electrophoresis

**Figure 2:**
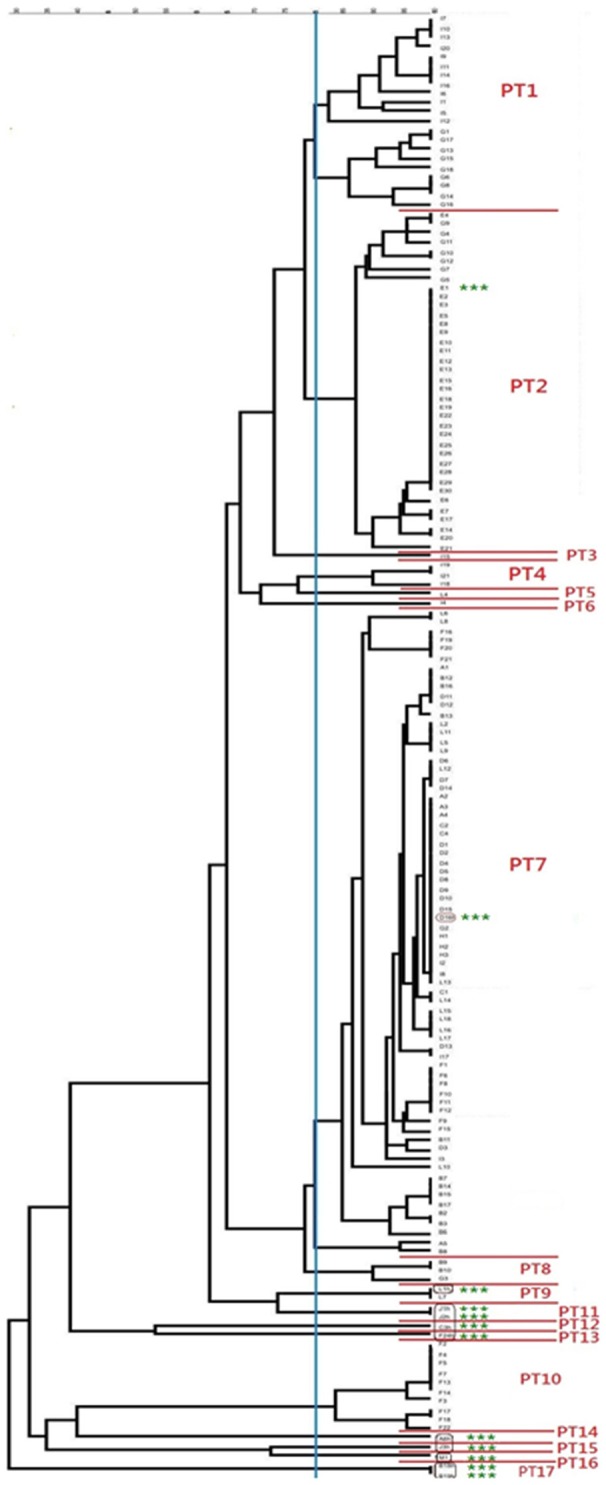
**Dendrogram showing the genetic relatedness of bovine and human ***Staphylococcus aureus*** isolates**. The vertical blue line shows the 80% similarity cut-off, whilst the pulsotypes are delineated by red lines and the human isolates are highlighted using green asterisks.

All *S. aureus* isolates could be typed using macrorestriction followed by PFGE. Digestion of genomic DNA with *Sma*I produced eight to 13 fragments which ranged in size from <76 kb to approximately 674 kb. The *S. aureus* isolates of bovine origin generated 58 electrophoretic patterns which grouped into 10 pulsotypes (PT1 to PT10) at an 80% similarity level (Figure 2). The majority of the bovine isolates, 93.2% (136/146), grouped into four major pulsotypes [PT1 (*n* = 21, 14.4%), PT2 (*n* = 36, 24.7%), PT7 (*n* = 69, 47.3%), and PT10 (*n* = 10, 6.8%)]. At five of the sampling sites (A, C, D, E, and H) only a single *S. aureus* pulsotype was recovered from the mastitic milk samples. Two *S. aureus* pulsotypes were identified at sites B and F, whilst four pulsotypes were identified amongst the isolates from site G and five *S. aureus* pulsotypes were identified at site I. Three *S. aureus* isolates (E/CPS1h; D/CPS16h; and L/CPS1h) recovered from human nasal swabs were assigned to pulsotypes common to bovine isolates namely PT2, PT7, and PT9. The nine remaining human isolates were allocated to seven separate pulsotypes (PT11–PT17).

### Genotyping of *Staphylococcus aureus* isolates

The 16 representative *S. aureus* isolates of bovine origin were assigned to 11 different *spa* types which varied in length between 1 and 11 repeats. Four new *spa* types, t15533 (*n* = 1), t15536 (*n* = 1), t15538 (*n* = 1) and t15539 (*n* = 4) were identified among the bovine isolates in this study. Other *spa* types detected include: t015 (*n* = 1), t064 (*n* = 1), t189 (*n* = 1), t529 (*n* = 1), t693 (*n* = 1), t730 (*n* = 1), and t1201 (*n* = 3). Among the ten representative human *S. aureus* isolates a diverse range of *spa* types were detected including: t012 (*n* = 1), t015 (*n* = 1), t064 (*n* = 1), t189 (*n* = 1), t360 (*n* = 1), t581 (*n* = 1), t1201 (*n* = 1), t1476 (*n* = 1), t2172 (*n* = 1), and t7023 (*n* = 1). MLST of isolates revealed a limited number of predominant sequence types among the 16 bovine isolates typed with ST352 (CC97) (*n* = 6) and ST2992 (CC97) (*n* = 5) being the most common. Other sequence types detected include: ST8 (CC8) (*n* = 1), ST97 (CC97) (*n* = 1), ST351 (CC705) (*n* = 1), ST508 (CC45) (*n* = 1) and a novel sequence type, ST3538 (*n* = 1) with an allelic profile of 357-1-378-1-1-5-3. The human isolates typed as ST5 (CC5) (*n* = 1), ST8 (CC8) (*n* = 2), ST15 (CC15) (*n* = 1), ST30 (CC30) (*n* = 1), ST45 (CC45) (*n* = 1); ST97 (CC97) (*n* = 1), ST352 (CC97) (*n* = 1), ST508 (C45) (*n* = 1), and ST707 (CC707) (*n* = 1). The distribution of the different *S. aureus* sequence types and *spa* types are correlated with the pulsotypes in Table 3.

## Discussion

To investigate the specific objectives of this study a cross-section of farming operations were sampled including commercial dairy herds, government managed agriculture college herds and communal animals. The sampling approach ensured the collection of a diverse range of *S. aureus* isolates for characterization. One hundred and forty six bovine *S. aureus* isolates and 12 human isolates were characterized and genotyped using a combination of PCR and M-PCR assays, *agr* typing and PFGE, with representative isolates being evaluated further using *spa* typing and MLST.

### Virulence gene profiling of bovine and human *Staphylococcus aureus*

Hemolysin genes were identified in all *S. aureus* isolates with bovine and human isolates testing positive for at least alpha- and delta-hemolysin. The beta-hemolysin gene was detected in the majority (87%) of the bovine isolates. The high occurrence of *hlb* among bovine isolates is in agreement with other reports (Zecconi et al., 2006; Monecke et al., 2007; Ikawaty et al., 2010). Comparatively, *hlb* was less frequently detected in human isolates. It is pertinent to relate the detection of *hlb* to the presence of the IEC genes, which are located on β-hemolysin converting bacteriophages (van Wamel et al., 2006), as the occurrence of IEC genes is accompanied by insertional inactivation of *hlb*. The IEC genes are considered a hallmark of human *S. aureus* isolates, although it has been demonstrated that not all human isolates carry these genes (Verkaik et al., 2011). In this study *sak* was detected in 67% (8/12) of the human isolates examined. This is not particularly high but may simply be a consequence of the limited number of human isolates evaluated in this study. Comparatively, *sak* was only sporadically detected among the bovine mastitis isolates in this study with 13% (19/146) of isolates found to carry the gene. With the exception of one of the 19 isolates, the *hlb* gene was not detected in any of the *sak*-positive bovine isolates.

All bovine isolates evaluated were positive for *luk*ED, this is consistent with the results of other studies which have reported a high prevalence of *luk*ED among bovine mastitis isolates (Fueyo et al., 2005; Monecke et al., 2007). Interesting to note was the distribution of *luk*M which was predominantly detected among isolates from two of the major bovine pulsotypes namely PT7 and PT10. Rainard et al. (2003) demonstrated that *luk*M was highly cytotoxic for bovine neutrophils leading the researchers to conclude that isolates producing this toxin would have a selective advantage in the bovine host enabling the bacteria to overcome the host immune system and establish infection (Rainard et al., 2003; Hata et al., 2006, 2010; Vrieling et al., 2015). This observation has been verified *in vivo* where Haveri et al. (2007) found *luk*M-positive *S. aureus* strains more likely to be associated with clinical mastitis cases instead of subclinical infections.

All *S. aureus* isolates were screened for a comprehensive range of PTSAg genes including the genes encoding the classical enterotoxins, enterotoxin A [SEA] to enterotoxin E [SEE], the newer enterotoxins [SEG, SEH, SEI], enterotoxin-like proteins [SElJ, SElK, SElL, SElM, SElN, SElO, SElP, SElQ SElR, SElU] and the toxic shock syndrome toxin-1 [TSST-1]. Although approximately 27% of the bovine *S. aureus* isolates in this study tested positive for one or more PTSAg genes, all of these isolates originated from only three herds namely, E, F, and L. The *sec*-*sell* gene combination was detected in the majority of the bovine isolates originating from herd E whilst in herd F the *sec* and *sell* genes presented together with *seg, seli, seln, selu*, and *tst*. Many of the genes encoding PTSAgs reside on mobile genetic elements, which accounts for the co-occurrence of particular genes as well as the horizontal dissemination of genes amongst compatible bacterial strains residing in the same ecological niche. The *sec* and *sell* genes have been shown to reside on the pathogenicity island SaPI3 (Fitzgerald et al., 2001; Holtfreter and Broeker, 2005) whilst the combination of *sec*-*sell* together with *tst* is characteristic of SaPI2 (Fitzgerald et al., 2001; Smyth et al., 2005). Enterotoxin C is recognized as an important virulence factor in bovine mastitis and SEC-producing *S. aureus* strains have been associated with cases of severe mastitis (Matsunaga et al., 1993; Ferens et al., 1998; Haveri et al., 2007).

The carriage of PTSAgs by *S. aureus* isolates recovered from cases of bovine mastitis has varied considerably between studies (Srinivasan et al., 2006; Haveri et al., 2007; Wang et al., 2009; de Freitas Guimarães et al., 2013). In addition to *S. aureus* strain variation, which may be influenced by environmental and management factors, the observed differences could also be attributable to herd selection criteria or the geographic location of the study (Srinivasan et al., 2006). One study conducted in the United States reported the occurrence of one or more PTSAgs in more than 93% of the *S. aureus* isolates investigated (Srinivasan et al., 2006). The high prevalence led the investigators to suggest that PTSAgs are important in the development and, or, maintenance of intramammary infections. Given the immunosuppressive effect PTSAgs have on the host's immune system it is certainly a reasonable conclusion. In general data from other studies have reported much lower PTSAg gene prevalences with figures ranging from 10 to 70% (Haveri et al., 2007; Oliveira et al., 2011; de Freitas Guimarães et al., 2013). Overall the wide range in prevalences suggest that PTSAgs may provide *S. aureus* strains with a competitive advantage but these toxins are not essential for the development of mastitis (Wang et al., 2009; de Freitas Guimarães et al., 2013). It is apparent that the exact role of each toxin in the pathogenesis of staphylococcal mastitis still requires clarification.

The low occurrence of PTSAgs detected among the bovine *S. aureus* isolates evaluated in this study should also be considered in terms of the public health impact. The milk from sub-clinical cases of mastitis will enter into the milk supply chain together with PTSAg-positive *S. aureus* strains. The risk of bacterial transfer to workers in the supply chain or the ingestion of raw or incorrectly pasteurized milk is likely to be minimal in light of the low prevalence of PTSAg gene-positive *S. aureus* detected in this study.

### Diversity of bovine isolates within herds and relation to global clonal lineages

The number of *S. aureus* pulsotypes causing IMIs in the dairy herds investigated ranged from one to five, with one (*n* = 5) or two (*n* = 2) pulsotypes being detected in the majority of herds. The most common pulsotype PT7, was detected in 75% (9/12) of the herds sampled. The limited genetic diversity observed within herds is consistent with the clonal nature of *S. aureus* and the contagious, lateral spread of the bacterium between cows in close contact (Capurro et al., 2010). The *S. aureus* isolates recovered from two herds, G and I, comprised of four and five *S. aureus* pulsotypes respectively. The owner of herd G indicated that cows and replacement heifers were sourced from other herds, in addition to rearing his own calves. This practice could account for the larger number of *S. aureus* pulsotypes observed amongst the mastitis isolates recovered from this herd. Middleton et al. (2002) reported that herds that used replacement heifers had more strains of *S. aureus* than closed herds which supplemented their dairy herds with their own stock. The same reason cannot be used to explain the high number of *S. aureus* pulsotypes detected in Herd I as the owner reportedly maintains a closed herd and rears his own replacement heifers. Other factors which are believed to influence genetic diversity and which may be contributing factors in this herd, include, herd size, milk parlor practices and specific management practices including treatment protocols and policies regarding the segregation and, or, the culling of *S. aureus* positive cows (Middleton et al., 2002; Middleton, 2013).

MLST of isolates belonging to the predominant bovine pulsotype PT7, and the second major pulsotype, PT2, identified the strains as ST352 (CC97) and ST97 (CC97) respectively. Both sequence types have been reported extensively from bovine mastitis cases in many countries including, but not limited to, Brazil, Chile, Italy, Japan, Norway, Spain, the Netherlands and the United States (Smith et al., 2005; Rabello et al., 2007; Hata et al., 2010). The expansive distribution of this successful lineage suggests that the strains are specifically adapted to the bovine host (Smith et al., 2005; Budd et al., 2015).

MLST of representative isolates from PT1, PT3, PT4, and PT6 were found to have a sequence type corresponding to *S. aureus* ST2992. Interestingly only a single report on the occurrence of *S. aureus* ST2992, a single-locus variant (SLV) of ST97, could be found in the literature. This ST was previously reported as a carriage isolate from a moose in a Polish study (http://saureus.mlst.net). All ST2992 isolates in this study presented with novel *spa* types. A novel *S. aureus* sequence type, ST3538, was identified from a milk sample collected at sampling site L. The sequence type, which also presented with a novel *spa* type, is a SLV of ST2992 (CC97). The detection of a new *S. aureus* sequence type is further evidence supporting the diversification of CC97.

The *S. aureus* isolates comprising PT10 originated from a single herd, sampling site F, and were genotyped as ST351 (CC705). This cluster of isolates is interesting for a number of reasons. Firstly, these isolates were the only bovine *S. aureus* isolates found to have a type II accessory gene regulator. All other bovine *S. aureus* isolates in this study were found to have a type I accessory gene regulator; a finding which is consistent with other reports (Gilot and van Leeuwen, 2004; Buzzola et al., 2007). It has been demonstrated that *S. aureus* strains belonging to *agr* group I can invade epithelial cells and persist in higher numbers in mammary gland tissue than strains belonging to other *agr* groups (Buzzola et al., 2007), suggesting a functional adaptation to the udder milieu. The second point of interest regarding PT10/ST351 isolates is that all 10 isolates tested negative using a commercial slide agglutination test which is used for the rapid identification of *S. aureus* isolates. Stutz et al. (2011) reported a similar observation with bovine mastitis isolates when using a different commercial kit which was based on the same biological principles. The researchers attributed the negative results to genetic polymorphisms in the clumping factor (*clf* A) and fibronectin-binding (*fnb*A) genes as well as a premature stop codon in the *spa* gene. Subsequent genotyping of the isolates by Moser et al. (2013) found all isolates to be ST151 (CC151). *Staphylococcus aureus* ST351 is a double locus variant of ST151 and it would be interesting to establish if the molecular basis behind the agglutination-negative result is due to similar genetic polymorphisms. The practical implication of the false-negative test results reported with the latex agglutination test kit is quite considerable, especially in mastitis diagnostic laboratories relying on the assay to identify presumptive *S. aureus* isolates (Pyörälä et al., 2011; Stutz et al., 2011).

### Genotypic relatedness of bovine and human *Staphylococcus aureus* isolates and investigation of transmission between hosts

From the 13 sampling sites, *S. aureus* was simultaneously recovered from bovine milk and human nasal swabs at seven of the sites. The diversity and genetic relatedness of the bovine and human *S. aureus* isolates were initially examined through the use of PFGE. In comparison to the bovine isolates, the *S. aureus* strains recovered from close human contacts were far more genetically diverse. Nine of the 12 human isolates clustered separately from the bovine isolates in eight separate pulsotypes (PT11 to PT17) and comprised of multilocus sequence types not evident among the bovine isolates including ST5, ST15, ST30, ST45, and ST707.

At three of the sampling sites, D, E, and L, the *S. aureus* isolates recovered from both of the hosts were genetically indistinguishable. At sites D and E, the predominant bovine strains detected, PT7/ST352/CC97/t1201 and PT2/ST97/CC97/t189 respectively were also recovered from human nasal swabs collected at the sites. *Staphylococcus aureus* ST97 strains have been reported from human carriage and clinical specimens (Lozano et al., 2011; Udo et al., 2011; Spoor et al., 2013). The presence of virulence markers such as *hlb, luk*ED, and *luk*M, which, although not exclusively found in bovine-associated strains, but are known to provide a selective advantage in the bovine host, suggest that the *S. aureus* isolates are bovine in origin. So does the lack of IEC-associated genes. Unfortunately, due to the fact that only a single round of sampling was done it was not possible to establish in the above cases whether the human contacts were transiently or permanently colonized with this *S. aureus* strain.

At sampling site L, genetically indistinguishable *S. aureus* strains belonging to PT9/ST8/CC8/t064 were recovered from both milk and human nasal swabs. The majority of the bovine isolates collected from this site were ST352; no other *S. aureus* ST8 isolates were detected. *Staphylococcus aureus* ST8 has been reported extensively from human carriage and clinical specimens (Sakwinska et al., 2009; Oosthuysen et al., 2014). This lineage also comprises a number of significant MRSA clones (Monecke et al., 2011). Whilst ST8 has been documented from cases of bovine mastitis (Sakwinska et al., 2011) the prevailing context in this herd, suggests the *S. aureus* isolate is of human origin and has been transferred to the bovine host.

The close contact between dairy cows and humans, particularly in the milking parlor creates the opportunity for the transmission of bacteria between hosts. Whilst evidence suggests that some *S. aureus* strains are adapted to colonize and infect certain host species, other lineages are non-specific. Irrespective hereof, *S. aureus* is a versatile pathogen and is readily able to adapt to new environments through gene mutation, transfer and decay (Spoor et al., 2013). It is primarily for this reason that the interface between animal populations and humans needs to be monitored in order to allow for changes in population dynamics to be timeously detected.

The low number of human *S. aureus* isolates recovered from the nasal swabs of close human contacts is considered one of the primary limitations of this study. The availability of only 12 human *S. aureus* isolates restricted the comparisons which could be made between isolates originating from bovine and human hosts. Further sampling at additional sites in KZN and other provinces of South Africa will potentially provide a larger number of isolates for characterization and will enable the preliminary observations made in this study to be confirmed.

## Conclusion

This study presents the first insight into the virulence characteristics and genetic diversity of *S. aureus* strains causing mastitis in dairy herds in a province of South Africa. The absence of clinically significant antimicrobial resistance genes and virulence markers, such as the Panton Valentine leukocidin encoding gene (*pvl*), together with the low occurrence of PTSAg genes among bovine isolates suggests that the human and public health risks posed by the bovine *S. aureus* populations are low. Genotyping of bovine isolates revealed a limited number of clones with several new *spa* types and a new *S. aureus* sequence type being documented. A greater genetic diversity was observed among the human isolates evaluated in this study. The detection of genotypically indistinguishable *S. aureus* isolates from bovine and human hosts at three of the sampling sites is suggestive of bacterial transmission and supports the need for vigilant monitoring of *S. aureus* populations at the interface between dairy cows and humans.

## Author contributions

TS, ME, and MK conceived and designed the study. TS collected samples and performed all laboratory analyses. TS and MK analyzed the genotyping data. TS wrote the manuscript with critical appraisal and contributions received from all of the authors. All authors read and approved the final version of the manuscript.

## Funding

The authors would like to thank the University of Pretoria, National Health Laboratory Services, RESCOM, the National Research Foundation (NRF) Research Technology Fund (RTF14011560804) and the KZN Department of Agriculture and Rural Development for financial support.

### Conflict of interest statement

The authors declare that the research was conducted in the absence of any commercial or financial relationships that could be construed as a potential conflict of interest.

## References

[B1] Akineden Ö. Annemüller C. Hassman A. A. Lämmler C. Wolter W. Zschöck M. (2001). Toxin genes and other characteristics of *Staphylococcus aureus* isolates from milk of cows with mastitis. Clin. Diag. Lab. Immunol. 8, 959–964. 10.1128/cdli.8.5.959-964.2001 11527811 PMC96179

[B2] Argudín M. Á. Mendoza M. C. Rodicio M. R. (2010). Food poisoning and *Staphylococcus aureus* enterotoxins. Toxins 2, 1751–1773. 10.3390/toxins2071751 22069659 PMC3153270

[B3] Becker K. Skov R. L. von Eiff C. (2015). Staphylococcus, Micrococcus, and other catalase-positive cocci, in Manual of Clinical Microbiology, 11th Edn, eds Jorgensen J. H. Pfaller M. A. Carroll K. C. Funke G. Landry M. L. Richter S. S. (Washington, DC: ASM Press), 354–382.

[B4] Ben Ayed S. Boutiba-Ben Boubaker I. Ennigrou S. Ben Redjeb S. (2008). Accessory gene regulator (agr) typing of *Staphylococcus aureus* isolated from human infections. Archs. Inst. Pasteur Tunis 85, 1–4. 19469411

[B5] Budd K. E. McCoy F. Monecke S. Cormica P. Mitchell J. Keane O. M. (2015). Extensive genomic diversity among bovine-adapted *Staphylococcus aureus*: evidence for a genomic rearrangement within CC97. PLoS ONE 10:e0134592. 10.1371/journal.pone.0134592 26317849 PMC4552844

[B6] Buzzola F. R. Alvarez L. P. Tuchscherr L. P. N. Barbagelata M. S. Lattar S. M. Calvinho L.. (2007). Differential abilities of capsulated and noncapsulated *Staphylococcus aureus* isolates from diverse *agr* groups to invade mammary epithelial cells. Infect. Immun. 75, 886–891. 10.1128/IAI.01215-06 17145949 PMC1828494

[B7] Capurro A. Aspán A. Ericsson Unnerstad H. Persson Waller K. Artursson K. (2010). Identification of potential sources of *Staphylococcus aureus* in herds with mastitis problems. J. Dairy Sci. 93, 180–191. 10.3168/jds.2009-2471 20059917

[B8] Clark N. C. Cooksey R. C. Hill B. C. Swenson J. M. Tenover F. C. (1993). Characterization of glycopeptide-resistant enterococci from U.S. hospitals. Antimicrob. Agents Chemother. 37, 2311–2317. 10.1128/AAC.37.11.2311 8285611 PMC192384

[B9] Cuny C. Wieler L. H. Witte W. (2015). Livestock-associated MRSA: the impact on humans. Antibiotics 4, 521–543. 10.3390/antibiotics4040521 27025639 PMC4790311

[B10] de Freitas Guimarães F. Nóbrega D. B. Bodelão Richini-Pereira V. B. Marson P. M. de Figueiredo J. C. P. Langoni P.. (2013). Enterotoxin genes in coagulase-negative and coagulase-positive staphylococci isolated from bovine milk. J. Dairy Sci. 96, 1–7. 10.3168/jds.2012-5864 23477822

[B11] Enright M. C. Day N. P. Davies C. E. Peacock S. J. Spratt B. G. (2000). Multilocus sequence typing for characterization of methicillin-resistant and methicillin-susceptible clones of *Staphylococcus aureus*. J. Clin. Microbiol. 38, 1008–1015. 10698988 10.1128/jcm.38.3.1008-1015.2000 PMC86325

[B12] Faria N. A. Carrico J. A. Oliveira D. C. Ramirez M. de Lencastre H. (2008). Analysis of typing methods for epidemiological surveillance of both methicillin-resistant and methicillin-susceptible *Staphylococcus aureus* strains. J. Clin. Microbiol. 46, 136–144. 10.1128/JCM.01684-07 17989188 PMC2224309

[B13] Feil E. Cooper J. Grundmann H. Robinson D. Enright M. Berendt T.. (2003). How clonal is *Staphylococcus aureus*? J. Bacteriol. 185, 3307–3316. 10.1128/JB.185.11.3307-3316.2003 12754228 PMC155367

[B14] Ferens W. A. Davis W. C. Hamilton M. J. Park Y. H. Deobald C. F. Fox L.. (1998). Activation of bovine lymphocyte subpopulations by staphylococcal enterotoxin C. Infect. Immun. 66, 573–580. 9453611 10.1128/iai.66.2.573-580.1998 PMC107943

[B15] Ferry T. Perpoint T. Vandenesch F. Etienne J. (2005). Virulence determinants in *Staphylococcus aureus* and their involvement in clinical syndromes. Curr. Infect. Dis. Rep. 7, 420–428. 10.1007/s11908-005-0043-8 16225779

[B16] Feßler A. Scott C. Kadlec K. Ehricht R. Monecke S. Schwarz S. (2010). Characterization of methicillin-resistant *Staphylococcus aureus* ST398 from cases of bovine mastitis. J. Antimicrob. Chemoth. 65, 619–625. 10.1093/jac/dkq021 20164198

[B17] Fitzgerald J. R. (2012). Livestock-associated *Staphylococcus aureus*: origin, evolution and public health threat. Trends Microbiol. 20, 192–198. 10.1016/j.tim.2012.01.006 22386364

[B18] Fitzgerald J. R. Sturdevant D. E. Mackie S. M. Gill S. R. Musser J. M. (2001). Evolutionary genomics of *Staphylococcus aureus*: insights into the origin of methicillin-resistant strains and the toxic shock syndrome epidemic. Proc. Natl. Acad. Sci. U.S.A. 98, 8821–8826. 10.1073/pnas.161098098 11447287 PMC37519

[B19] Fueyo J. M. Mendoza M. C. Rodicio M. R. Muñiz J. Alvarez M. A. Martín M. C. (2005). Cytotoxin and pyrogenic toxin superantigen gene profiles of *Staphylococcus aureus* associated with subclinical mastitis in dairy cows and relationships with macrorestriction genomic profiles. J. Clin. Microbiol. 43, 1278–1284. 10.1128/jcm.43.3.1278-1284.2005 15750096 PMC1081256

[B20] Gilot P. Lina G. Cochard T. Poutrel B. (2002). Analysis of the genetic variability of genes encoding the RNA III-activating components Agr and TRAP in a population of *Staphylococcus aureus* strains isolated from cows with mastitis. J. Clin. Microbiol. 40, 4060–4067. 10.1128/JCM.40.11.4060-4067.2002 12409375 PMC139642

[B21] Gilot P. van Leeuwen W. (2004). Comparative analysis of *agr* locus diversification and overall genetic variability among bovine and human *Staphylococcus aureus* isolates. J. Clin. Microbiol. 42, 1265–1269. 10.1128/JCM.42.3.1265-1269.2004 15004090 PMC356838

[B22] Gordon R. Lowy F. C. (2008). Pathogenesis of methicillin-resistant *Staphylococcus aureus* infection. Clin. Infect. Dis. 46, 350–359. 10.1086/533591 18462090 PMC2474459

[B23] Graveland H. Duim B. van Duijkeren E. Heederik D. Wagenaar J. A. (2011). Livestock-associated methicillin-resistant *Staphylococcus aureus* in animals and humans. Int. J. Med. Microbiol. 301, 630–634. 10.1016/j.ijmm.2011.09.004 21983338

[B24] Graves L. M. Swaminathan B. (2001). PulseNet standardized protocol for subtyping *Listeria monocytogenes* by macrorestriction and pulsed-field gel electrophoresis. Int. J. Food Microbiol. 65, 55–62. 10.1016/S0168-1605(00)00501-8 11322701

[B25] Haenni M. Galofaro L. Ponsin C. Bes M. Laurent F. Madec J. Y. (2010). Staphylococcal bovine mastitis in France: enterotoxins, resistance and the human Geraldine methicillin-resistant *Staphylococcus aureus* clone. J. Antimicrob. Chemother 66, 216–218. 10.1093/jac/dkq417 21071460

[B26] Harmsen D. Claus H. Witte W. Rothgänger J. Claus H. Turnwald D.. (2003). Typing of methicillin-resistant *Staphylococcus aureus* in a university hospital setting by using novel software for *spa* repeat determination and database management. J. Clin. Microbiol. 41, 5442–5448. 10.1128/JCM.41.12.5442-5448.2003 14662923 PMC309029

[B27] Hata E. Katsuda K. Kobayashi H. Nishimori K. Uchida I. Higashide M.. (2008). Bacteriological characteristics of *Staphylococcus aureus* isolates from humans and bulk milk. J. Dairy Sci. 91, 564–569. 10.3168/jds.2007-0457 18218742

[B28] Hata E. Katsuda K. Kobayashi H. Ogawa T. Endo T. Eguchi M. (2006). Characteristics and epidemiologic genotyping of *Staphylococcus aureus* isolates from bovine mastitic milk in Hoddaido, Japan. J. Vet. Med. Sci. 68, 165–170. 10.1292/jvms.68.165 16520540

[B29] Hata E. Katsuda K. Kobayashi H. Uchida I. Tanaka K. Eguchi M. (2010). Genetic variation among *Staphylococcus aureus* strains from bovine milk and their relevance to methicillin-resistant isolates from humans. J. Clin. Microbiol. 48, 2130–2139. 10.1128/JCM.01940-09 20392913 PMC2884479

[B30] Haveri M. Hovinen M. Roslöf A. Pyörälä S. (2008). Molecular types and genetic profiles of *Staphylococcus aureus* strains isolated from bovine intramammary infections and extramammary sites. J. Clin. Microbiol. 46, 3728–3735. 10.1128/JCM.00769-08 18799704 PMC2576574

[B31] Haveri M. Roslöf A. Rantala L. Pyörälä S. (2007). Virulence genes of bovine *Staphylococcus aureus* from persistent and nonpersistent intramammary infections with different clinical characteristics. J. App. Microbiol. 103, 993–1000. 10.1111/j.1365-2672.2007.03356.x 17897203

[B32] Haveri M. Taponen S. Vuopio-Varkila J. Salmenlinna S. Pyörälä S. (2005). Bacterial genotype affects the manifestation and persistence of bovine *Staphylococcus aureus* intramammary infection. J. Clin. Microbiol. 43, 959–961. 10.1128/JCM.43.2.959-961.2005 15695718 PMC548092

[B33] Holtfreter S. Broeker B. M. (2005). Staphylococcal superantigens: do they play a role in sepsis? Arch. Immunol. Ther. Exp. 53, 13–27. 15761373

[B34] Ikawaty R. Brouwer E. C. van Duijkeren E. Mevius D. Verhoef J. Fluit A. C. (2010). Virulence factors of genotyped bovine mastitis *Staphylococcus aureus* isolates in the Netherlands. Int. J. Dairy Sci. 5, 60–70. 10.3923/ijds.2010.60.70

[B35] Jarraud S. Mougel C. Thioulouse J. Lina G. Meugnier H. Forey F.. (2002). Relationships between *Staphylococcus aureus* genetic background, virulence factors, *agr* groups (alleles), and human disease. Infect. Immun. 70, 631–641. 10.1128/IAI.70.2.631-641.2002 11796592 PMC127674

[B36] Joo Y. S. Fox L. K. Davis W. C. Bohach G. A. Park Y. H. (2001). *Staphylococcus aureus* associated with mammary glands of cows: genotyping to distinguish different strains among herds. Vet. Microbiol. 80, 131–138. 10.1016/S0378-1135(00)00381-3 11295333

[B37] Juhász-Kaszanyitzky É. Jánosi S. Somogyi P. Dán Á. van der Graaf-van Bloois L. van Duijkeren E.. (2007). MRSA transmission between cows and humans. Emerg. Infect. Dis. 13, 630–632. 10.3201/eid1304.060833 17553285 PMC2725960

[B38] Kadlec K. Wendlandt S. Feβler A. T. Schwarz S. (2015). Methods for the detection of antimicrobial resistance and the characterization of *Staphylococcus aureus* isolates from food-producing animals and food of animal origin in Antimicrobial Resistance and Food Safety, eds Chen C.-Y. Yan X. Jackson C. R. (Chennai: Academic Press), 207–232.

[B39] Kapur V. Sischo W. Greer R. Whittam T. Musser J. (1995). Molecular population genetic analysis of *Staphylococcus aureus* recovered from cow. J. Clin. Microbiol. 33, 376–380. 7714195 10.1128/jcm.33.2.376-380.1995 PMC227951

[B40] Le Loir Y. Baron F. Gautier M. (2003). *Staphylococcus aureus* and food poisoning. Gen. Mol. Res. 2, 63–76. 12917803

[B41] Lindsay J. A. Holden M. T. (2004). *Staphylococcus aureus*: superbug, super genome? Trends Microbiol. 12, 378–385. 10.1016/j.tim.2004.06.004 15276614

[B42] Lowy F. (2013). Staphylococcal infections, in Harrison's Principles of Internal Medicine, eds Fauci A. Braunwald E. Casper D. Hauser S. Longo D. Jameson J. (New York, NY: The McGraw-Hill Companies Inc.), 386–399.

[B43] Lozano C. Gómez-Sanz E. Benito D. Aspiroz C. Zarazaga M. Torres C. (2011). *Staphylococcus aureus* nasal carriage, virulence traits, antibiotic resistance mechanisms, and genetic lineages in healthy humans in Spain, with detection of CC398 and CC97 strains. Int. J. Med. Microbiol. 301, 500–505. 10.1016/j.ijmm.2011.02.004 21570348

[B44] Lundberg Å. Aspán A. Nyman A. Ericsson Unnerstad H. Persson Waller K. (2014). Associations between bacterial genotype and outcome of bovine clinical *Staphylococcus aureus* mastitis. Acta Vet. Scand. 56:2. 10.1186/1751-0147-56-2 24397927 PMC3901377

[B45] Matsunaga T. Kamata S. Kakiichi N. Uchida K. (1993). Characteristics of *Staphylococcus aureus* isolated from peracute, acute and chronic bovine mastitis. J. Vet. Med. Sci. 55, 297–300. 10.1292/jvms.55.297 8513013

[B46] McClure J.-A. Conly J. M. Lau V. Elsayed S. Louie T. Hutchins W.. (2006). Novel multiplex PCR assay for detection of Staphylococcal virulence marker Panton-Valentine leukocidin genes and simultaneous discrimination of methicillin-susceptible from –resistant staphylococci. J. Clin. Microbiol. 44, 1141–1144. 10.1128/JCM.44.3.1141-1144.2006 16517915 PMC1393128

[B47] McDougal L. K. Steward C. D. Killgore G. E. Chaitram J. M. McAllister S. K. Tenover F. C. (2003). Pulsed-field gel electrophoresis typing of oxacillin-resistant *Staphylococcus aureus* isolates from the United States: establishing a national database. J. Clin. Microbiol. 41, 5113–5120. 10.1128/JCM.41.11.5113-5120.2003 14605147 PMC262524

[B48] Mehrotra M. Wang G. Johnson W. M. (2000). Multiplex PCR for detection of genes for *Staphylococcus aureus* enterotoxins, exfoliative toxins, toxic shock syndrome toxin 1, and methicillin resistance. J. Clin. Microbiol. 38, 1032–1035. 10698991 10.1128/jcm.38.3.1032-1035.2000 PMC86330

[B49] Middleton J. R. (2013). *Staphylococcus aureus*: have we learned anything in the last 50 years? in Proceedings of the National Mastitis Council (NMC) Regional Meeting 2013. Available online at: http://www.nmconline.org/articles/staphaureus50.pdf [Accessed 8 July 2016].

[B50] Middleton J. R. Fox L. K. Gay J. M. Tyler J. W. Besser T. E. (2002). Use of pulsed-field gel electrophoresis for detecting differences in *Staphylococcus aureus* strain populations between dairy herds with different cattle importation practices. Epidemiol. Infect. 129, 387–395. 10.1017/S095026880200746X 12403115 PMC2869898

[B51] Monecke S. Coombs G. Shore A. C. Coleman D. C. Akpaka P. Borg M.. (2011). A field guide to pandemic, epidemic and sporadic clones of methicillin-resistant *Staphylococcus aureus*. PLoS ONE 6:e17936. 10.1371/journal.pone.0017936 21494333 PMC3071808

[B52] Monecke S. Kuhnert P. Hotzel H. Slickers P. Ehricht R. (2007). Microarray based study on virulence-associated genes and resistance determinants of *Staphylococcus aureus* isolates from cattle. Vet. Microbiol. 125, 128–140. 10.1016/j.vetmic.2007.05.016 17614219

[B53] Moser A. Stephan R. Corti S. Johler S. (2013). Comparison of genomic and antimicrobial resistance features of latex agglutination test-positive and latex agglutination test-negative *Staphylococcus aureus* isolates causing bovine mastitis. J. Dairy Sci. 96, 329–334. 10.3168/jds.2012-5944 23127911

[B54] Oliveira L. Rodrigues A. C. Hulland C. Ruegg P. L. (2011). Enterotoxin production, enterotoxin gene distribution, and genetic diversity of *Staphylococcus aureus* recovered from milk of cows with subclinical mastitis. Am. J. Vet. Microbiol. 72, 1361–1368. 10.2460/ajvr.72.10.1361 21962279

[B55] Omoe K. Hu D. L. Takahashi-Omoe H. Nakane A. Shinagawa K. (2005). Comprehensive analysis of classical and newly described staphylococcal superantigenic toxin genes in *Staphylococcus aureus* isolates. FEMS Microbiol. Lett. 246, 191–198. 10.1016/j.femsle.2005.04.007 15899405

[B56] Omoe K. Ishikawa M. Shimoda Y. Hu D.-L. Ueda S. Shinagawa K. (2002). Detection of *seg, seh*, and *sei* genes in *Staphylococcus aureus* isolates and determination of the enterotoxin productivities of *S. aureus* isolates harbouring seg, seh, or sei genes. J. Clin. Microbiol. 40, 857–862. 10.1128/JCM.40.3.857-862.2002 11880405 PMC120241

[B57] Oosthuysen W. F. Orth H. Lombard C. J. Sinha B. Wasserman E. (2014). Population structure analyses of *Staphylococcus aureus* at Tygerberg hospital, South Africa, reveals a diverse population, a high prevalence of Panton-Valentine leukocidin genes, and unique local methicillin-resistant *S. aureus clones*. Clin. Microbiol. Infect. 20, 652–659. 10.1111/1469-0691.12452 24206111

[B58] Park J. Y. Fox L. K. Seo K. S. McGuire M. A. Park Y. H. Rurangirwa F. R.. (2011). Detection of classical and newly described staphylococcal superantigen genes in coagulase-negative staphylococci isolated from bovine intramammary infections. Vet. Microbiol. 147, 149–154. 10.1016/j.vetmic.2010.06.021 20667668 PMC3689430

[B59] Petrovski K. R. Trajcev M. Buneski G. (2006). A review of the factors affecting the costs of bovine mastitis. J. S. Afr. Vet. Assoc. 77, 52–60. 10.4102/jsava.v77i2.344 17120619

[B60] Plata K. Rosato A. E. Wegrzyn G. (2009). *Staphylococcus aureus* as an infectious agent: overview of biochemistry and molecular genetics of its pathogenicity. Acta Bio. Pol. 56, 597–612. 20011685

[B61] Pyörälä S. Simojoki H. Taponen S. (2011). News about mastitis-causing staphylococci, in Paper Presented at the European Buiatrics Forum, eds Maillard R. Navetat H. (Marseille: Societe Francaise de Buiatrie), 93–104.

[B62] Rabello R. F. Moreira B. M. Lopes R. M. Teixeira L. M. Riley L. W. Castro A. C. (2007). Multilocus sequence typing of *Staphylococcus aureus* isolates recovered from cows with mastitis in Brazilian dairy herds. J. Med. Microbiol. 56, 1505–1511. 10.1099/jmm.0.47357-0 17965353

[B63] Rainard P. Corrales J. C. Barrio M. B. Cochard T. Poutrel B. (2003). Leucotoxic activities of *Staphylococcus aureus* strains isolated from cows, ewes, and goats with mastitis: importance of LukM/LukF'-PV leukotoxin. Clin. Diag. Lab. Immunol. 10, 272–277. 10.1128/cdli.10.2.272-277.2003 12626454 PMC150537

[B64] Sakwinska O. Giddey M. Moreillon M. Morisset D. Waldvogen A. Moreillon P. (2011). *Staphylococcus aureus* host range and human-bovine host shift. Appl. Environ. Microbiol. 77, 5908–5915. 10.1128/AEM.00238-11 21742927 PMC3165375

[B65] Sakwinska O. Kuhn G. Balmelli C. Francioli P. Giddey M. Perreten V.. (2009). Genetic diversity and ecological success of *Staphylococcus aureus* strains colonizing humans. Appl. Environ. Microbiol. 75, 175–183. 10.1128/AEM.01860-08 18978084 PMC2612194

[B66] Schmidt T. Kock M. M. Ehlers M. M. (2015). Diversity and antimicrobial susceptibility profiling of staphylococci isolated from bovine mastitis cases and close human contacts. J. Dairy Sci. 98, 6256–6269. 10.3168/jds.2015-9715 26188567

[B67] Shopsin B. Gomez M. Montgomery S. O. Smith D. H. Waddington M. Dodge D. E.. (1999). Evaluation of protein A polymorphic region DNA sequencing for typing of *Staphylococcus aureus* strains. J. Clin. Microbiol. 37, 3556–3563. 10523551 10.1128/jcm.37.11.3556-3563.1999 PMC85690

[B68] Smeltzer M. S. Lee C. Y. Harik N. Hart M. E. (2009). Molecular basis of pathogenicity, in Staphylococci in Human Disease, 2nd Edn, eds Crossley K. B. Jefferson K. Archer G. L. Fowler V. G. (Singapore: Wiley-Blackwell), 65–108.

[B69] Smith E. M. Green L. E. Medley G. F. Bird H. E. Fox L. K. Schukken Y. H.. (2005). Multilocus sequence typing of intercontinental bovine *Staphylococcus aureus* isolates. J. Clin. Microbiol. 43, 4737–4743. 10.1128/JCM.43.9.4737-4743.2005 16145135 PMC1234155

[B70] Smyth D. S. Feil E. J. Meaney W. J. Hartigan P. J. Tollersrud T. Fitzgerald J. R.. (2009). Molecular genetic typing reveals further insights into the diversity of animal-associated *Staphylococcus aureus*. J. Med. Microbiol. 58, 1343–1353. 10.1099/jmm.0.009837-0 19528163

[B71] Smyth D. S. Hartigan P. J. Meaney W. J. Fitzgerald J. R. Deobald C. F. Bohach G. A.. (2005). Superantigen genes encoded by the *egc* cluster and SaPIbov are predominant among *Staphylococcus aureus* isolates from cows, goats, sheep, rabbits and poultry. J. Med. Microbiol. 54, 401–411. 10.1099/jmm.0.45863-0 15770028

[B72] Sommerhäuser J. Kloppert B. Wolter W. Zschöck M. Sobiraj A. Failing K. (2003). The epidemiology of *Staphylococcus aureus* infections from subclinical mastitis in dairy cows during a control programme. Vet. Microbiol 96, 91–102. 10.1016/S0378-1135(03)00204-9 14516711

[B73] Spoor L. E. McAdam P. R. Weinert L. A. Rambaut A. Hasman H. Aarestrup F. M.. (2013). Livestock origin for a human pandemic clone of community-associated methicillin-resistant *Staphylococcus aureus*. mBio 4:e00356–13. 10.1128/mBio.00356-13 23943757 PMC3747577

[B74] Srinivasan V. Sawant A. A. Gillespie B. E. Headrick S. J. Ceasaris L. Oliver S. P. (2006). Prevalence of enterotoxin and toxic shock syndrome toxin genes in *Staphylococcus aureus* isolated from milk of cows with mastitis. Foodborne Path. Dis. 3, 274–283. 10.1089/fpd.2006.3.274 16972776

[B75] Stegger M. Andersen P. S. Kearns A. Pichon B. Holmes M. A. Edwards G.. (2012). Rapid detection, differentiation and typing of methicillin-resistant *Staphylococcus aureus* harbouring either *mecA* or the new *mecA* homologue *mecA*_*LGA*251_. Clin. Microbiol. Infect. 18, 395–400. 10.1111/j.1469-0691.2011.03715.x 22429460

[B76] Stutz K. Stephan R. Tasara T. (2011). SpA, ClfA, and FnbA genetic variations lead to Staphaurex test-negative phenotypes in bovine mastitis *Staphylococcus aureus* isolates. J. Clin. Microbiol. 49, 638–646. 10.1128/JCM.01148-10 21147952 PMC3043514

[B77] Sung J. M. Lloyd D. H. Lindsay J. A. (2008). *Staphylococcus aureus* host specificity: comparative genomics of human versus animal isolates by multi-strain microarray. Microbiology 154, 1949–1959. 10.1099/mic.0.2007/015289-0 18599823

[B78] Tenhagen B. A. Vossenkuhl B. Käsbohrer A. Alt K. Kraushaar B. Guerra B.. (2014). Methicillin-resistant *Staphylococcus aureus* in cattle food chains – prevalence, diversity, and antimicrobial resistance in Germany. J. Anim. Sci. 92, 2741–2751. 10.2527/jas.2014-7665 24778337

[B79] Tenover F. C. Arbeit R. D. Goering R. V. Mickelsen P. A. Murray B. E. Persing D. H.. (1995). Interpreting chromosomal DNA restriction patterns produced by pulsed-field gel electrophoresis: criteria for bacterial strain typing. J. Clin. Microbiol. 33, 2233–2239. 7494007 10.1128/jcm.33.9.2233-2239.1995 PMC228385

[B80] Trees E. Rota P. A. MacCannell D. Gerner-Smidt P. (2015). Molecular epidemiology, in Manual of Clinical Microbiology, 11th Edn, eds Jorgensen J. H. Pfaller M. A. Caroll K. C. Funke G. Landry M. L. Richter S. S. (Washington, DC: ASM Press), 131–160

[B81] Udo E. E. Aly N. Y. A. Sarkhoo E. Al-Sawan R. Al-Asar A.-S. M. (2011). Detection and characterization of an ST97-SCCmec-V community-associated methicillin-resistant *Staphylococcus aureus* clone in a neonatal intensive care unit and special care baby unit. J. Med. Microbiol. 60, 600–604. 10.1099/jmm.0.028381-0 21292856

[B82] van Wamel W. J. B. Rooijakkers S. H. M. Ruyken M. van Kessel K. P. M. van Strijp J. A. G. (2006). The innate immune modulators staphylococcal complement inhibitor and chemotaxis inhibitory protein of *Staphylococcus aureus* are located on β-hemolysin-converting bacteriophages. J. Bacteriol. 188, 1310–1315. 10.1128/JB.188.4.1310-1315.2006 16452413 PMC1367213

[B83] Verkaik N. J. Benard M. Boelens H. A. de Vogel C. P. Nouwen J. L. Verbrugh H. A. (2011). Immune evasion cluster-positive bacteriophages are highly prevalent among human *Staphylococcus aureus strains*, but they are not essential in the first stages of nasal colonization. Clin. Microbial. Infect. 17, 343–348. 10.1111/j.1469-0691.2010.03227.x 20370801

[B84] Vesterholm-Nielsen M. Olhom Larsen M. Olsen J. E. Moller Aarestrup F. (1999). Occurrence of *blaZ* gene in penicillin resistant *Staphylococcus aureus* isolated from bovine mastitis in Denmark. Acta Vet. Scand. 40, 279–286. 10605145 10.1186/BF03547026 PMC8043211

[B85] Vrieling M. Koymans K. J. Heesterbeek D. A. C. Aerts P. C. Rutten V. P. M. G. de Haas C. J.. (2015). Bovine *Staphylococcus aureus* secretes the leukocidin LukMF' to kill migrating neutrophils through CCR1. mBio 6:e00335–15. 10.1128/mBio.00335-15 26045537 PMC4462618

[B86] Wang S.-C. Wu C.-M. Xia S.-C. Qi Y.-H. Xia L.-N. Shen J.-Z. (2009). Distribution of superantigenic toxin genes in *Staphylococcus aureus* isolates from milk samples of bovine subclinical mastitis cases in two major diary production regions of China. Vet. Microbiol. 137, 276–281. 10.1016/j.vetmic.2009.01.007 19217725

[B87] Wright J. Novick R. (2003). Virulence mechanisms in MRSA pathogenesis, in MRSA: Current Perspectives, eds Fluit A. Schmitz F.-J. (Wymondham: Caister Academic Press), 213–240.

[B88] Yamaguchi T. Nishifuji K. Sasaki M. Fudaba Y. Aepfelbacher M. Takata T.. (2002). Identification of the *Staphylococcus aureus etd* pathogenicity island which encodes a novel exfoliative toxin, ETD, and EDIN-B. Infect. Immun. 70, 5835–5845. 10.1128/IAI.70.10.5835-5845.2002 12228315 PMC128317

[B89] Zadoks R. N. van Leeuwen W. B. Kreft D. Fox L. K. Barkema H. W. Schukken Y. H.. (2002). Comparison of *Staphylococcus aureus* isolates from bovine and human skin, milking equipment, and bovine milk by phage typing, pulsed-field gel electrophoresis, and binary typing. J. Clin. Microbiol. 40, 3894–3902. 10.1128/JCM.40.11.3894-3902.2002 12409348 PMC139627

[B90] Zecconi A. Cesaris L. Liandris E. Daprà V. Piccinini R. (2006). Role of several *Staphylococcus aureus* virulence factors on the inflammatory response in the bovine mammary gland. Microb. Pathog. 40, 177–183. 10.1016/j.micpath.2006.01.001 16517115

